# Surgical management of Glioma Grade 4: technical update from the neuro-oncology section of the Italian Society of Neurosurgery (SINch®): a systematic review

**DOI:** 10.1007/s11060-023-04274-x

**Published:** 2023-03-24

**Authors:** Tamara Ius, Giovanni Sabatino, Pier Paolo Panciani, Marco Maria Fontanella, Roberta Rudà, Antonella Castellano, Giuseppe Maria Vincenzo Barbagallo, Francesco Belotti, Riccardo Boccaletti, Giuseppe Catapano, Gabriele Costantino, Alessandro Della Puppa, Francesco Di Meco, Filippo Gagliardi, Diego Garbossa, Antonino Francesco Germanò, Maurizio Iacoangeli, Pietro Mortini, Alessandro Olivi, Federico Pessina, Fabrizio Pignotti, Giampietro Pinna, Antonino Raco, Francesco Sala, Francesco Signorelli, Silvio Sarubbo, Miran Skrap, Giannantonio Spena, Teresa Somma, Carmelo Sturiale, Filippo Flavio Angileri, Vincenzo Esposito

**Affiliations:** 1 Division of Neurosurgery, Head-Neck and NeuroScience Department, University Hospital of Udine, Udine, Italy; 2 Institute of Neurosurgery, Fondazione Policlinico Gemelli, Catholic University, Rome, Italy; 3 Unit of Neurosurgery, Mater Olbia Hospital, Olbia, Italy; 4 Division of Neurosurgery, Department of Surgical Specialties, Radiological Sciences and Public Health, University of Brescia, Brescia, Italy; 5 Department of Neuro-Oncology, University of Turin and City of Health and Science Hospital, 10094 Torino, Italy; 6 Neurology Unit, Hospital of Castelfranco Veneto, 31033 Castelfranco Veneto, Italy; 7 Department of Neuroradiology, San Raffaele Scientific Institute, Vita-Salute University, Milan, Italy; 8 Department of Medical and Surgical Sciences and Advanced Technologies (G.F. Ingrassia), Neurological Surgery, Policlinico “G. Rodolico - San Marco” University Hospital, University of Catania, Catania, Italy; 9 Interdisciplinary Research Center On Brain Tumors Diagnosis and Treatment, University of Catania, Catania, Italy; 10 Neurosurgery Operative Unit, University Hospital of Sassari, Sassari, Italy; 11 Division of Neurosurgery, Department of Neurological Sciences, Ospedale del Mare, Naples, Italy; 12 Division of Neurosurgery, ARNAS Civico Hospital, Palermo, Italy; 13 Neurosurgical Clinical Department of Neuroscience, Psychology, Pharmacology and Child Health, Careggi Hospital, University of Florence, Florence, Italy; 14 Department of Neurosurgery, Fondazione IRCCS Istituto Neurologico Carlo Besta, Milan, Italy; 15 Department of Pathophysiology and Transplantation, University of Milan, Milan, Italy; 16 Johns Hopkins Medical School, Baltimore, MD USA; 17 Department of Neurosurgery and Gamma Knife Radiosurgery, San Raffaele Scientific Institute, Vita-Salute University, Milan, Italy; 18 Department of Neuroscience “Rita Levi Montalcini,” Neurosurgery Unit, University of Turin, Torino, Italy; 19 Division of Neurosurgery, BIOMORF Department, University of Messina, Messina, Italy; 20 Department of Neurosurgery, Università Politecnica Delle Marche, Azienda Ospedali Riuniti, Ancona, Italy; 21 Department of Biomedical Sciences, Humanitas University, Via Rita Levi Montalcini 4, 20090 Milan, Italy; 22 Neurosurgery Department, IRCCS Humanitas Research Hospital, Via Manzoni 56, 20089 Milan, Italy; 23 Unit of Neurosurgery, Department of Neurosciences, Hospital Trust of Verona, 37134 Verona, Italy; 24 Division of Neurosurgery, Department of NESMOS, AOU Sant’Andrea, Sapienza University, Rome, Italy; 25 Department of Neurosciences, Biomedicines and Movement Sciences, Institute of Neurosurgery, University of Verona, 37134 Verona, Italy; 26 Department of Basic Medical Sciences, Neuroscience and Sense Organs, Neurosurgery Unit, University “Aldo Moro”, 70124 Bari, Italy; 27 Department of Neurosurgery, Santa Chiara Hospital, Azienda Provinciale Per I Servizi Sanitari (APSS), Trento, Italy; 28 Neurosurgery Unit, Fondazione IRCCS Policlinico S. Matteo, Pavia, Italy; 29 Division of Neurosurgery, Department of Neurosciences, Reproductive and Odontostomatological Sciences, Università Degli Studi Di Napoli Federico II, Naples, Italy; 30 Neurosurgery Operative Unit, Bellaria Hospital, Bologna, Italy; 31 Department of Neurosurgery “Giampaolo Cantore”-IRCSS Neuromed, Pozzilli, Italy; 32 Department of Human, Neurosciences-”Sapienza” University of Rome, Rome, Italy

**Keywords:** Glioma, Extent of resection, Intraoperative neurophysiological monitoring, Surgical planning, Navigated transcranial magnetic stimulation (nTMS), Intraoperative imaging

## Abstract

**Purpose:**

The extent of resection (EOR) is an independent prognostic factor for overall survival (OS) in adult patients with Glioma Grade 4 (GG4). The aim of the neuro-oncology section of the Italian Society of Neurosurgery (SINch®) was to provide a general overview of the current trends and technical tools to reach this goal.

**Methods:**

A systematic review was performed. The results were divided and ordered, by an expert team of surgeons, to assess the Class of Evidence (CE) and Strength of Recommendation (SR) of perioperative drugs management, imaging, surgery, intraoperative imaging, estimation of EOR, surgery at tumor progression and surgery in elderly patients.

**Results:**

A total of 352 studies were identified, including 299 retrospective studies and 53 reviews/meta-analysis. The use of Dexamethasone and the avoidance of prophylaxis with anti-seizure medications reached a CE I and SR A. A preoperative imaging standard protocol was defined with CE II and SR B and usefulness of an early postoperative MRI, with CE II and SR B. The EOR was defined the strongest independent risk factor for both OS and tumor recurrence with CE II and SR B. For intraoperative imaging only the use of 5-ALA reached a CE II and SR B. The estimation of EOR was established to be fundamental in planning postoperative adjuvant treatments with CE II and SR B and the stereotactic image-guided brain biopsy to be the procedure of choice when an extensive surgical resection is not feasible (CE II and SR B).

**Conclusions:**

A growing number of evidences evidence support the role of maximal safe resection as primary OS predictor in GG4 patients. The ongoing development of intraoperative techniques for a precise real-time identification of peritumoral functional pathways enables surgeons to maximize EOR minimizing the post-operative morbidity.

## Introduction

The annual incidence of gliomas is approximately of six cases per 100,000 people [1], with a slight prevalence in men. While the majority of cases are sporadic, it is estimated that about 5% of gliomas show a hereditary component in rare tumor predisposition syndromes (Cowden’s Syndrome, Turcot’s Syndrome, Lynch’s Syndrome, Li Fraumeni’s Syndrome and Neurofibromatosis type I and II) [2–5]. Characteristics of clinical onset are widely variable, including new-onset epilepsy, focal deficits, neurocognitive impairment, and symptoms and signs of increased intracranial pressure. Incidental diagnosis is extremely rare [6].

Advances in molecular testing and genomic analysis implies a continuous identification of subgroups with different prognosis. As a result of these advances, the 2021 WHO Classification gathers as adult Glioma Grade 4 (GG4) both the Astrocytomas IDH-mutant Grade 4 and the astrocytoma IDH wild-type, which in turn currently defines the Glioblastoma (GBM) class [7].

Recent integrative studies showed that patients diagnosed with these tumors have variable prognosis influenced not only by the molecular profile but also by the resection degree achieved [8–11].

In this clinical setting, different prognostic factors have been suggested, including age, extent of resection  (EOR), size of necrosis, and specific molecular markers [i.e., MGMT methylation (O6 -methylguanine-DNA methyl-transferase)], mutation of IDH1, IDH2 (isocitrate dehydrogenase) or TERT (telomerase reverse transcriptase), 1p19q codeletion, overexpression of EGFR (epidermal growth factor receptor), PDGFRA(Platelet-derived growth factor receptor alpha) [12, 13].

Compelling evidence, based on objective tumoral volume analysis, supports the role of EOR in GG4 patients as one of the main predictive survival factors [14–18]. Surgical treatment, however, can rarely be considered as radical, due to infiltrating nature, multifocal presentation, and ill-defined tumor margins [9]. Despite years of molecular discoveries and technological advances surgery, followed by radiotherapy (RT) and concomitant and adjuvant chemotherapy (CT) with temozolomide (TMZ) (Stupp protocol), still represents the current standard of care [11, 19].

Contemporary technological and conceptual innovations have thus improved the safety of surgical resection, while expanding the surgical options and indications for GG4 surgical treatment [20, 21]. Several techniques currently used during surgery, such as intraoperative ultrasound (iUS), cortical mapping, sodium fluorescein [22] and 5-ALA fluorescence (5-aminolevulinic acid), tend to favor higher rates of total resection, with apparent increased survival [17, 18, 23].

Unfortunately, the infiltrative growing, the rapid proliferative rate of malignant cells, and the appearance of treatment-resistant cell clones shortly after initial therapy, tend to promote tumor relapse, within 2 cm of resection margins, regardless the EOR [24].

**Fig. 1:**
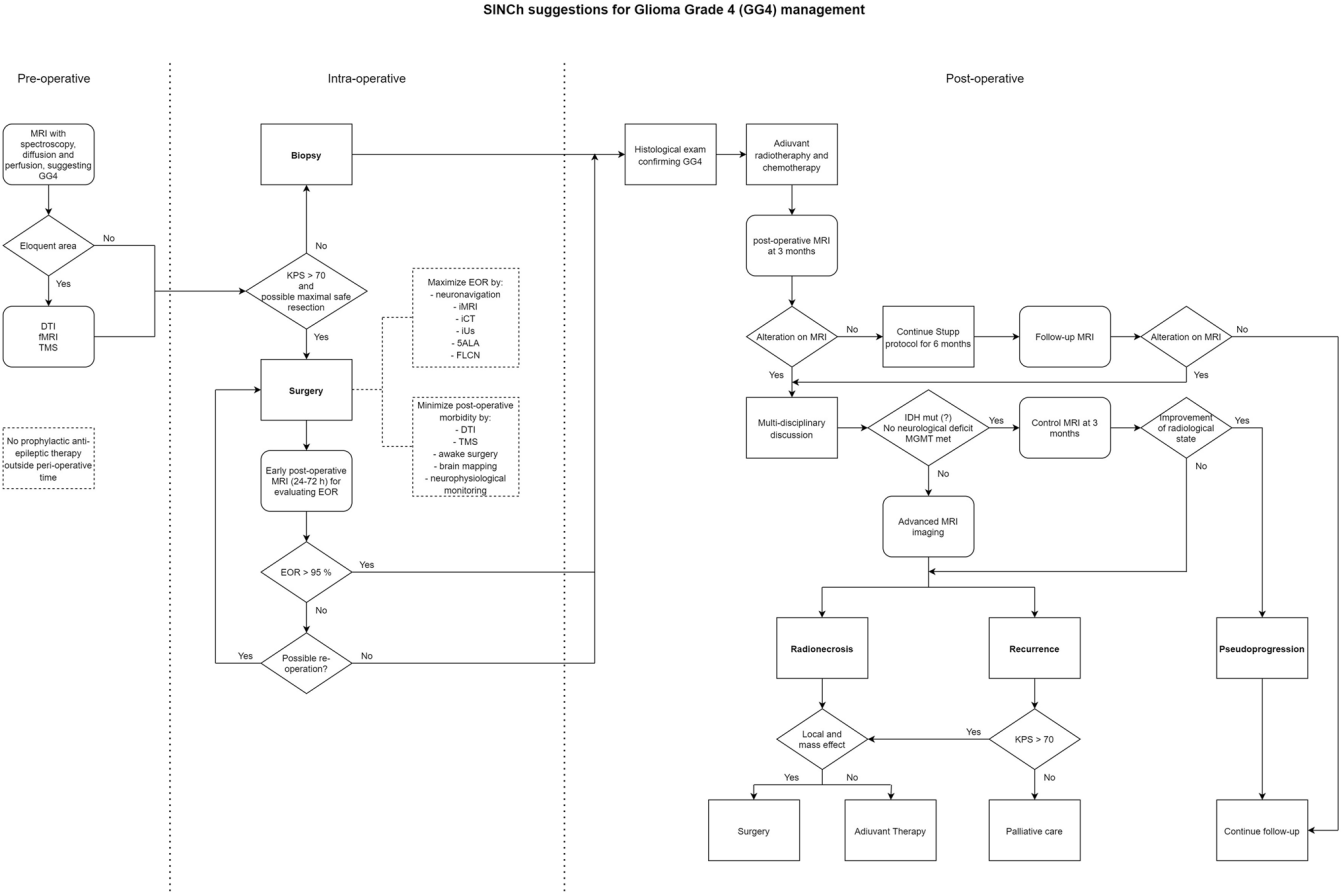
GG4 management algorithm proposed by SINch

Technological advances are thus in continuous development to improve surgical tools and methods, with the goal of optimizing the EOR beyond the radiological borders, when functionally possible. Considering recent technical advantages, the aim of this paper is to provide a general overview of the current trends and technical tools that are available in the management of GG4 surgery (Fig. 1).

The advantages and limitations are highlighted and discussed in compliance with the maximal safe resection principle in glioma surgery. The role of the major treatment modalities of surgery was revised in terms of accuracy and safety.

In addition, the preoperative use of antiepileptic and steroids are discussed according to the current literature.

## Materials and methods

The methods used in this systematic review were prespecified and are presented in accordance with the 2020 Preferred Reporting Items for Systematic Reviews and Meta-Analyses (PRISMA) guidelines. A literature search was performed using the electronic databases of Ovid MEDLINE(R) Epub Ahead of Print, In-Process & Other Non-Indexed Citations, Ovid MEDLINE(R) Daily and Ovid MEDLINE(R) (1946 to Present [September 2022]). A top-up search was subsequently performed with the same databases: Ovid MEDLINE(R) Epub Ahead of Print, In-Process & Other Non-Indexed Citations, Ovid MEDLINE(R) Daily and Ovid MEDLINE(R) (1946 to September 24, 2022), with a filter for articles published from 2018 onwards. Medical Subject Heading (MeSH) terms “high grade glioma” “glioblastoma” [MeSH] AND “surg*” [MeSH] and free text terms: “extent of resection” OR “surgery” OR “survival” OR “outcome” OR “surgical planning” OR “preoperative planning” OR “radiotherapy” OR “elderly” OR “intraoperative monitoring” OR “IDH mutation” OR “1p/19q codeletion” OR “tumor grade” OR “MGMT methylation” OR “chemotherapy” OR “adjuvant” OR “recurrence”, were used to ensure the search was as comprehensive as possible. The search strategy that was created, combined the two broad content areas of HGGs, evaluating investigation on intraoperative tools and surgical management.

These two content areas were combined using the Boolean operator “and”. Reference lists of identified studies were also reviewed to identify additional relevant studies.

### Inclusion criteria

To be eligible for inclusion in the review, the manuscripts identified had to: report primary data; include adult patients with GG4; and be published in English language. Although the focus of this review is on patients with GG4, the search strategy was deliberately broad to include a range of brain tumors in order to ensure all studies incorporating patients with gliomas, including studies with mixed pathologies (different types of brain tumors). If there was uncertainty about whether a manuscript was relevant or not, it was decided to include it for full-text review.

### Exclusion criteria

The following search results were excluded from this systematic review:

- Review papers, including systematic reviews, meta-analyses, and narrative reviews;
- Single patient case reports (case series or case studies with more than one patient were included);
- Dissertation abstracts;
- Book chapters/books;
- Studies focusing on children, without a predominantly adult population.

### Screening process

Manuscript titles were initially screened by 4 authors medically qualified specialists in neurosurgery to identify potentially relevant articles (F.P., F.B., G.C., T.S.). Then, abstracts of screened studies were screened independently by (T.I., P.P.P., G.S., S.S.) to identify relevant studies. Where ambiguity regarding eligibility persisted, the full article was reviewed and disagreements were resolved by consensus.

### Data extraction process

Data from studies meeting our inclusion criteria were extracted using a standardized data extraction proforma and critically appraised. The relevant information extracted from the manuscripts included: study setting; study population, participant demographics and baseline characteristics; details of intervention and control conditions, where applicable; study methodology; recruitment and study completion rates; outcomes and times of measurement.

Due to the wide variations in study design and outcome measures, it was not possible to perform a meta-analysis.

**Fig. 2:**
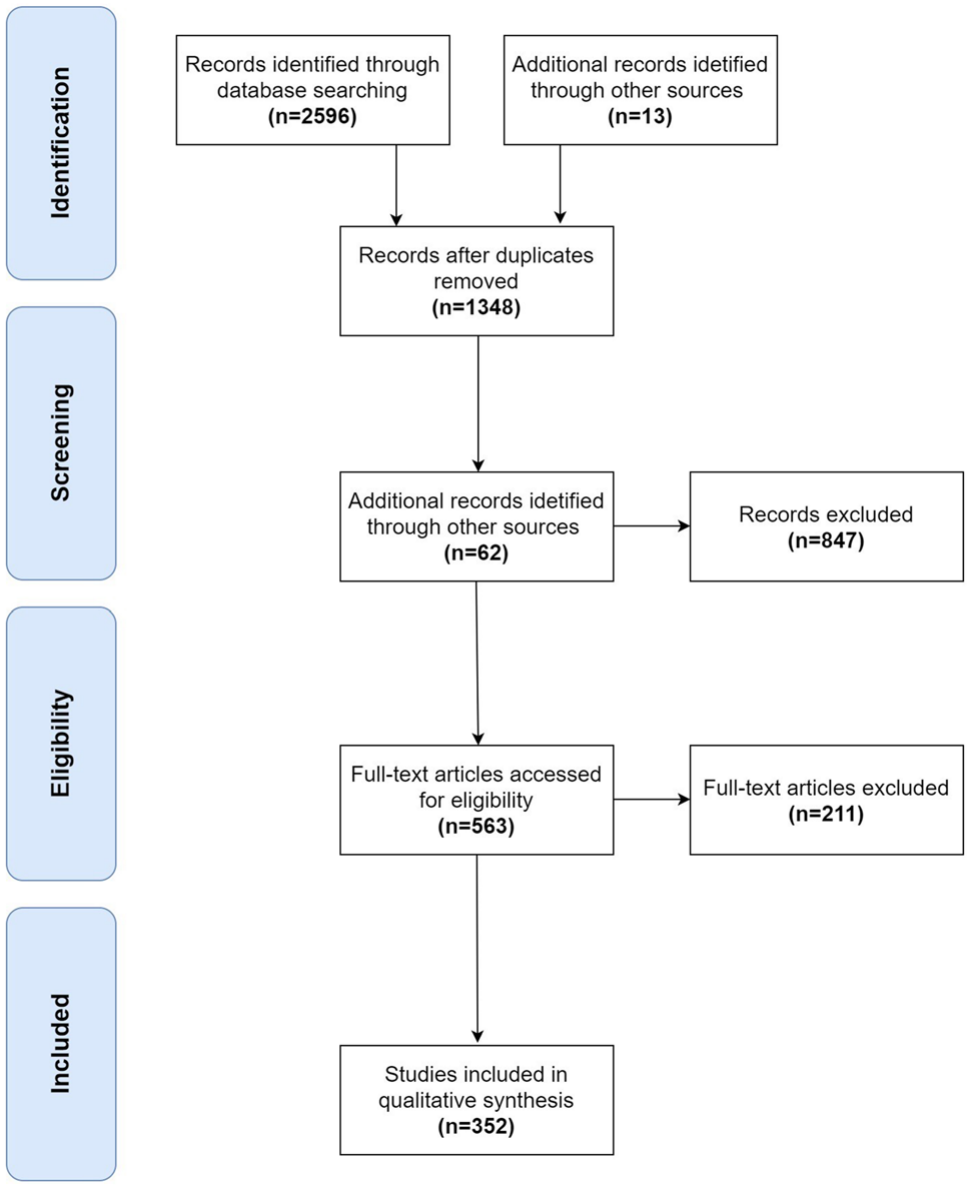
Flowchart of study search and selection

A total of 352 potentially relevant studies were identified, including 299 retrospective studies and 53 reviews/metanalysis (Fig. 2).

The final reference list was then checked by M.M.F., F.F.A., G.S. and F.P. All the authors divided papers to assess several statements as reported in Table 1 and in particular:

**Table 1:** Summary of Class of Evidence and Strength of Recommendation

Statement	Class of Evidence	Level of Recommendation
*Perioperative drugs management*		
Glioma patients who never suffered seizures should not be treated with primary prophylaxis with antiseizure medications (ASMs) [25–29]	I	A
Dexamethasone is considered the standard agent due to its high potency and relative lack of mineralocorticoid activity reduce the potential for fluid retention [30–32]	I	A
Anticoagulation with low molecular weight heparin or direct oral anticoagulants for established venous tromboembolism is recommended in patients with primary brain tumors [33]	II	B
*Imaging*		
The standard protocol includes anatomical, two-dimensional T2-weighted and FLAIR sequences (slice thickness ≤ 4 mm), FLAIR sequences (three-dimensional FLAIR is suggested as an alternative to 2D FLAIR) and three-dimensional T1-weighted images acquired pre- and post-contrast administration [34–36]	II	C
An early postoperative MRI is strongly suggested and should be performed within 48 to 72 hours after surgery, including DWI sequences [37]	II	B
Advanced techniques, such as diffusion MRI (dMRI), perfusion-weighted imaging (PWI), proton magnetic resonance spectroscopy (1HMRS) and positron emission tomography (PET) can provide a visual depiction and quantitative measurement of the pathophysiologic characteristics of the tumor [38, 39] PET-CT can provide information about biology, differential diagnosis, delineation of tumor extent for surgical or RT planning, which can be also usefull in post-treatment surveillance (progression vs pseudoprogression) [237]	III	C
*Surgery*		
The extent of surgical resection (EOR) is a strongest independent risk factor for both overall survival (OS) and tumor recurrence [40–43]	II	B
Intraoperative neurophysiological monitoring is associated with lower risk of permanent postoperative deficits and a higher EOR of tumors in eloquent areas [44, 45]	II	B
Cortico-subcortical mapping is the most sensitive and specific technique for the identification of critical cortical hubs and white matter bundles [46, 47]	III	C
Awake surgery can be considered an option, mainly in young patients with lesions in the dominant hemisphere involving language, motor and somato-sensory areas [47–50]	III	C
*Intraoperative imaging*		
NN: neuro-navigation systems represent the most used intraoperative tool used by neurosurgeons during tumor excision. Based on preoperative imaging, and overlap with FMRI-DTI, neuro-navigation allows preoperative identification of eloquent regions and guides the surgeon during intraoperative mapping and tumor resection [51–56]	III	C
iCT: iCT helped to verify EOR and to identify and resect pathological tissue. iCT represents a feasible and effective alternative for intraoperative updates of the neuro-navigation system [11, 57]	III	C
iUS: iUS is a real-time, accurate and inexpensive imaging method for optimizing the EOR in neurosurgical interventions Despite being an operator-dependent method, iUS is associated with a greater EOR and improved PFS and OS in glioma patients [58–60]	III	C
iMRI: iMRI was found to be associated with higher resection rates compared to the neuro-navigated procedures, Unfortunately, the high cost and the need for structural changes in the operating room have limited, to date, the spread of intraoperative MRI [59–64]	III	C
5-ALA: 5-ALA is a metabolic tracer that allows the intraoperative distinction of the boundaries between healthy tissue and tumor near the infiltration zones, thus guiding the glioma resection with an improved intraoperative enhancing vision [65–72]	II	B
Fluorescence: sodium fluorescein (FLCN): The intraoperative guidance determined by FLCN allows to predict histopathological alterations both in areas with contrast enhancement and in the margins of infiltration of the cerebral parenchyma that do not present a neuroimaging contrast [60, 70, 73–76]	III	C
*Intraoperative treatment options*		
In newly diagnosed HGGs, CWs implantations should not be considered as first-line therapeutic option [77–81]	II	B
*Estimation of extent of tumor resection *		
The objective estimation of the extent of tumor resection is fundamental in planning postoperative adjuvant treatments, stratifying patients’ prognosis and monitoring tumor evolution over time in response to adjuvant treatments [37, 41, 43, 82, 83]	II	B
The volumetric analysis can be carried out by manual segmentation of the areas of interest or by using the so-called ellipsoid volume technique or software with automatic or manual segmentation [82, 84]	III	C
Stereotactic image-guided brain biopsy is the procedure of choice when an extensive surgical resection is not feasible, as in deep-seated or multifocal tumors, or if the patient has considerable comorbidities increasing the risk of perioperative morbidity or even mortality [85–87]	II	B
*Surgery at tumor progression*		
The role of second surgery at recurrence is not definitively validated and should be evaluated on individual basis [88–96]	III	C
*Surgery in elderly patients*		
Thorough evaluation and surgical selection of elderly glioma patients may lead to favorable survival benefit [97–99]	III	C

Perioperative drugs Management 3 statements, Imaging 3 statements, Surgery 4 statements, Intraoperative imaging 6 statements, Intraoperative treatment options 1 steatment, Estimation of extent of tumor resection 3 statements, Surgery at tumor progression 1 statement, Surgery in elderly patients 1 statement.

The identification of the statement was performed by M.M.F., A.C. and R.R.

Lastly, two group of authors (T.I., P.P.P., F.P., S.S. and F.F.A, G.S., F.B., V.E.) classified the class of evidence for each statement from Class I to IV, and recommendations were graded Level A (established as effective, data from *multiple RCT (randomized clinical trials) or meta-analyses*), Level B (probably effective, data from*: single RCT or large non-randomized studies*), and Level C (possibly effective, data from *consensus of opinion/experts, small studies, retrospective studies, registries*).

Where ambiguity regarding evidence or recommendation existed, the full process was reviewed and disagreements were resolved by consensus.

The evaluation of the evidence and strength of recommendations for surgical management of gliomas are summarized in Table 1.

## Perioperative drugs management

The GG4 clinical onset is extremely variable with subacute presentation, with neurological signs and symptoms progressing over days or weeks. A sudden onset is less common and associated to epileptic seizure or neurological deficit for intralesional haemorrhage.

The most common presenting symptoms include non-specific symptoms as progressive, fatigue or headache, new-onset epilepsy, focal neurologic signs and mental status alterations in combination with signs of increased intracranial pressure [100]. These symptoms are related to different factors: (1) Tumoral invasion of eloquent brain areas; (2) mass effect by the tumor itself; (3) surrounding tumoral edema [101].

Perioperative medical treatment with steroids, antiepileptic drugs, and antithrombotic prophylaxis is indicated for symptoms relief and prevention of complications. Regarding the antithrombotic prophylaxis the treatment is indicated only in the early postoperative period) [102–109].

Treatment of acute venous thromboembolism (VTE) should follow the same protocol as in non-brain tumors, although in high grade gliomas anticoagulant therapy seems to increase by three-fold the risk of hemorrhage, but only with 1% of fatal hemorrhages [110].

### Steroid treatment

Tumoral edema is generated by local blood–brain barrier disruption and increased permeability caused by neoangiogenesis [32]. The extent of edema on neuroimaging must be interpreted alongside clinical symptoms, as it not always requires symptomatic treatment. Symptoms related to progressive edema tend to progress with subacute onset and gradual worsening. Systemic glucocorticoids should be considered in all patients who have symptomatic peritumoral edema. The mechanism of action of glucocorticoids for control of vasogenic edema is not fully understood: they are supposed to downregulate the vascular endothelial growth factor (VEGF) and also inhibit production of interleukin 1 (IL-1) [111]. Dexamethasone is considered the standard agent due to its effectiveness and relative lack of mineralocorticoid activity with low potential for fluid retention [30, 112]. In addition, it can be administered orally or intravenously with a 1:1 conversion ratio [113, 114].

The anti-edema effect of dexamethasone is dose-dependent. The starting dose should be individualized based according to edema degree, severity of symptoms and patient weight [112, 115, 116], to optimize the balance between the maximal efficacy and minimum dose-related side effects 32. Clinical response, rather than radiographic changes, should guide treatments. Most patients improve symptomatically within hours and achieve a maximum benefit from within 24 to 72 h [32]. In general, headache tend to respond better and more quickly than focal deficits. Once patients have responded and stabilized clinically on a given dose, a gradual taper should be attempted [112]. Dose should be reduced every 3–4 days while assessing its efficacy to control symptoms [117]. Slower tapering is necessary after 2–3 weeks of treatment in order to reduce the risk of hypoadrenalism due to hypothalamic axis suppression [32]. Despite their beneficial effect, glucocorticoids are associated with a large number of well- known side effects [113, 118]. Three complications are of particular concern: gastrointestinal complications, steroid myopathy, and opportunistic infections such as Pneumocystis pneumonia. In addition, retrospective studies have suggested that the use of steroids may be correlated with decreased overall survival (OS) in glioma patients, independent of potential confounding factors such as tumor size and performance status [32, 119]. If allowed clinically, a maintenance Dexamethasone dose of less than 4 mg per day should be employed [32]. Patients who have undergone only biopsy might need a prolongation of steroids administration in particular when starting of radiotherapy.

### Antiseizure medications (ASMs)

Although GG4 are less epileptogenic than lower-grade gliomas, seizures are usually more difficult to control with common ASMs and drug resistance reported in 20% of cases [120–122].

Epileptogenesis in GG4is partially related to increased intracranial pressure, edema, hypoperfusion, and neoangiogenesis. In addition structural and functional changes in the peritumoral cortex, increased concentrations of cytokines, chemokines, and growth factors contribute both to epileptogenesis and tumor invasiveness [123]. It remains still unclear how the same mechanisms that control tumor behavior may regulate epileptogenesis and how they may influence each other.

Seizure tend to start as focal and may either remain focal or secondarily generalize. Epilepsy should not be considered barely a symptom: it is an important source of morbidity and mortality in patients with brain tumors, and the risk of recurrence is high [124]. Therefore, treatment with a first-line ASM monotherapy at the lowest effective dose (monitoring serum intervals, if available) is needed. ASMs with no or minimal hepatic enzyme-inducing or -inhibiting properties, such as levetiracetam, pregabalin, lamotrigine, lacosamide, topiramate, are generally preferred, since these agents have a more favourable safety profile [125–130]. Levetiracetam is generally well tolerated but can cause neuropsychiatric side effects, including irritability, agitation, and anxiety especially in patients with frontal lobe tumors [131]. Use of valproate may be associated with a higher rate of hematologic toxicity, leading to CT treatment delays in glioma patients [132]. The initial use of multidrug regimens should be avoided as it decreases the likelihood of compliance, provides a narrower therapeutic window, and is less cost effective. Single ASM treatment has fewer side effects also because drug interactions are avoided. Approximately 50% of patients respond adequately to a single ASM [120]. In case of recurrent seizures after initiation of therapy, doses of the initial agent should be escalated (monitoring serum concentrations) before switching drugs or adding a second agent. If adequate seizure control is not achieved, an alternative or adjunctive ASM should be prescribed. Lacosamide has been increasingly studied as a complementary ASM for HGGs with refractory epilepsy and is generally well tolerated [127, 133].

Twenty-four percent of glioma patients, treated with ASMs, experience side effects that need a change in or discontinuation of antiseizure drug therapy [134], including: rash (especially during RT [135]), drug interactions (e.g., cytochrome P450 induction, increase or decrease of metabolic enzymes for steroids or CT agents).

Prophylactic ASMs are generally not recommended in glioma patients without a history of seizure [25, 26, 136]. Nevertheless, ASM prophylaxis for patients undergoing surgery is advocated by some authors, especially in case of surgery planned with brain mapping. This approach is based on data from observational studies and a limited number of small randomized trials leading to conflicting results [137–146]. The incidence of postoperative seizures is low (8%) even without prophylactic ASMs, and the incidence of clinically significant seizures is even lower (3%). In contrast, routine administration of ASMs may be associated with significant side effects [142, 143]. If postoperative seizure prophylaxis is employed, ASMs should be gradually tapered beginning one to two weeks after surgery, and then discontinued in patients who remain seizure-free [134].

### Antithrombotic treatment

VTE is a common complication in patients with primary brain tumors, with up to 20% of patients per year having a VTE event. Risk factors include patient-related, treatment-related, tumor-related factors, laboratory parameters, and hemostatic biomarkers [147]. Regarding tumor histology, vaso-occlusive and prothrombotic contributions in high-grade gliomas could be underlying necrosis and hypoxia. Indeed, tumor cells pseudo-palisades suggest that this morphologic phenomenon is created by a tumor cell population actively migrating away from a central hypoxic region due to vaso-occlusion caused by intravascular thrombosis. Both vascular endothelial growth factor-induced vascular permeability to plasma coagulation factors and the increased neoplastic expression of tissue factor likely contribute to a prothrombotic state favoring intravascular thrombosis [148]. Furthermore, mutations in the IDH-1 gene correlate with a low incidence of VTE compared to IDH-wild type tumors. In addition, expression of the glycoprotein podoplanin (a platelet activator) on brain tumors was associated with both intratumoral thrombi and a high risk of VTE [147].

Patient-related factors include older age, obesity, dependent functional status (dependency for activities of daily living, and limb paresis. Treatment-related factors include surgery (especially biopsies), subtotal resection, use of corticosteroids, and anti-VEGF therapy. Laboratory parameters and hemostatic biomarkers correlated with a higher risk of VTE are high white blood cell count, low platelet count, high soluble P-selectin levels, elevated coagulation factor VIII activity, and increased D-dimer levels [147, 149].

In other studies, no association between the presence or size of enhancing tumor was not a contraindication for anticoagulation as no difference was found in patients with or without intracerebral hemorrage (ICH), as well as no correlation between the EOR and the incidence of ICH incidence was found [149].

From a clinical point of view, the management of patients with primary brain tumors and VTE is challenging. Anticoagulation is required to treat patients; however, it is associated with an increased risk of intracranial haemorrhage [147]. For the general cancer population, pharmacological thromboprophylaxis with low-molecular-weight heparin (LMWH) is recommended in hospitalized patients and in the perioperative setting [150]. As the risk of VTE remains high throughout the course of the disease, a phase III randomized placebo-controlled trial (the PRODIGE study) aimed at evaluating the efficacy and safety of primary thromboprophylaxis with LMWH for up to 12 months in patients with malignant glioma, but the study was terminated early without being able to draw significant conclusions. A trend toward a reduced risk of VTE with heparin (hazard ratio [HR] 0.51, 95% confidence interval [CI]: 0.19–1.4, *p* = 0.29) was seen; however, a trend toward increased risk of major bleeding after 12 months was observed with heparin (HR 4.2, 95% CI: 0.48–36, *p* = 0.22), and all major bleeds were ICH [102]. Mortality after 12 months was not different between groups. In a meta-analysis involving 539 anticoagulated patients, the authors found that the overall risk for ICH in patients with glioblastoma was more than three-fold higher when receiving anticoagulation in comparison to those who were not receiving anticoagulation; nevertheless, the overall incidence of fatal ICH in this meta-analysis was less than 1% [110]. In the absence of high-quality data, primary pharmacological thromboprophylaxis cannot be recommended for patients with malignant glioma beyond the postoperative period [151]. Another study by Le Rhun et al., including more than 1,000 patients with newly diagnosed glioblastoma, showed that patients with anticoagulants while on radio/chemotherapy had worse survival than patients who did not use them; but patients under anticoagulant therapy mainly used them because of prior VTE events [152]. A meta-analysis of ten randomized controlled studies including 1263 patients with primary brain tumors undergoing craniotomy reported that patients receiving unfractionated heparin alone had a stronger risk reduction in VTE than patients receiving placebo (RR = 0.27; 95% CI 0.1–0.73) and heparin with mechanical prophylaxis together showed a lower VTE risk than mechanical prophylaxis alone (RR = 0.61; 95% CI 0.46–0.82) [109, 149].

Therefore, based on currently available evidence, in the 2019 updated international clinical practice guidelines for the treatment and prophylaxis of VTE in cancer patients, the use of heparin commenced postoperatively for the prevention of VTE in patients with cancer and undergoing neurosurgery is recommended; primary pharmacological prophylaxis is not recommended for patients with brain tumors not undergoing neurosurgery [33]. This is consistent with the clinical guidance from the International Society on Thrombosis and Hemostasis where no pharmacological prophylaxis is recommended for outpatients with brain tumors [153]. The European Society of Anesthesiology recommends for patients undergoing craniotomy with a high risk of VTE including malignancy, the initiation of mechanical thromboprophylaxis with intermittent pneumatic compression preoperatively in addition to heparin postoperatively as soon as the bleeding risk is decreased; the thromboprophylaxis is recommended to be continued until discharge [154].

In the case of a VTE event, more-recently-updated guidelines for treating VTE in cancer patients already include the data from randomized controlled studies comparing heparin and direct oral anticoagulants, with the general consensus that anticoagulation should be established with low molecular weight heparin. Anticoagulation should be given for 6 months. Thereafter an individual evaluation should be carried out for each patient including the risk–benefit ratio and tumor activity [33, 155]. Limited data regarding anticoagulation in patients with primary brain tumors leads to uncertainty with regard to which therapy is best for each patient. Current guidelines give support in the treatment of VTE in cancer patients but rarely offer recommendations, especially for brain tumor patients [149]. The American Society of Clinical Oncology states in its recently-published guidelines that in patients with primary brain tumors and VTE, anticoagulation should be offered, but uncertainty remains regarding the choice of agent and patients most likely to benefit [149, 155]. Furthermore, limited safety data is available for the use of direct oral anticoagulants in patients with primary brain tumors [155].

In 2019, the international clinical practice guidelines for the treatment and prophylaxis of VTE in cancer patients recommended anticoagulation for established VTE in patients with primary brain tumors with LMWH or direct oral anticoagulants (grade 2B)^33^.

## Role of surgery and estimation of the EOR

Modern glioma surgery focuses on the optimal balance between maximal tumor removal and preservation of quality of life [156, 157]. To achieve this goal, considering the infiltrative tumor growing [158], a detailed and personalized anatomo-functional pre-operative planning is fundamental.

Recent investigations have demonstrating the importance of the volumetric estimation of EOR as predictor of survival [8, 159–164].

To reduce the risk of overestimating a residual tumor in consideration of an increased non-specific contrast intake EOR estimation is recommended by using postoperative MRI obtained within 48 h after surgery (at latest within 72 h) [165].

Volumetric image analysis using 3-dimensional measurements should be applied to accurately quantify entire tumour volumes. The volumetric analysis can be carried out by manual segmentation of the areas of interest (ROI, region of interest) on MR images with post-contrast T1, FLAIR or T2 sequences using the DICOM format. The following formula is used to estimate the EOR: “EOR = preoperative tumor volume − postoperative tumor volume/preoperative tumor volume”.

Alternatively, the so-called ellipsoid volume technique or software with automatic or manual segmentation can be used [84].

The extent of resection should be assessed within 24–48 hours of surgery through MRI (or CT if MRI is not possible), before and after contrast administration; MRI should include diffusion-weighted (DWI) sequences to enable the detection of perioperative ischemia, that eventually enhance after 48 h. If postoperative MRI is performed after 48 h, tissue enhancement may be misinterpreted as a residual tumor, hampering follow-up evaluations. A lesser extent of resection and larger post- surgical residual tumor volumes are negative prognostic factors across gliomas of all grades and subtypes [23, 43, 157, 160, 166–168]. The dilemma regarding the superior predictive value amongst these variables is still open in the field of neuro-oncology.

The definition EOR should include reduction of tumor volume, as a measurement of surgical efficacy, and residual tumor volume (RV), as a measurement of remaining tumor burden [169].

Robust retrospective analysis of prospective data from a randomized trial yielded level IIB evidence that the EOR, maximized by a different combination of intraoperative tools, is positively associated with the OS in GG4 patients [23, 67, 163, 166, 167, 170–172].

In most papers, resection of a GG4 means resection of the contrast-enhancing area. However, nomenclature for definition of EOR achieved in glioma surgery is not standardized yet. A recent review by Karschnia et al. defined six categories for EOR in supratentorial contrast-enhancing glioma: “*supramaximal resection*”, if beyond contrast-enhancing tumor borders (class of evidence III); “*complete resection*”, when EOR corresponds to 100% of contrast-enhancing tumor (class of evidence IIB); “*near total resection*”, if EOR is >  = 95% contrast-enhancing tumor and <  = 1 cm^3^ residual contrast-enhancing tumor (class of evidence IV); “*subtotal resection*”, in case of >  = 80% EOR of contrast-enhancing tumor and <  = 5 cm^3^ residual contrast-enhancing tumor (class of evidence IV); “*partial resection*” when EOR is between 1 and 79% contrast-enhancing tumor and/or > 5 cm^3^ residual contrast-enhancing tumor, for mass effect-related symptoms (class of evidence IV); “*biopsy*”, if there is no reduction of tumor volume and performed for tissue-based diagnosis (class of evidence IV) [37].

Traditionally gross total resection (GTR) of post-contrast T1-weighted MRI tumor has been shown to improve OS and progression free survival (PFS) in patients with newly diagnosed GBM compared to subtotal resection (STR) or biopsy in multiple large population studies [23, 166]. However, in recent years, an increasing number of studies have documented that any increase in EOR is correlated to higher OS and PFS. One of the first papers published by Lacroix on a series of patients with GBM showed that a macroscopic excision greater than 98% of the total lesion correlated with a prolonged survival up to 13 months [23], compared to 8 months in patients with lower EOR values [161].

The early volumetric retrospective investigations suggested that at least 70–78% of the contrast-enhancing tumor volume represented the ideal resection target for survival benefit [84, 118, 173].

In 2016, Brown et al. [43] published a systematic literature review on EOR studies conducted in adult patients with newly diagnosed supratentorial GBMs, including 37 studies, published over the last four decades, with suitable data for meta-analysis (41,117 patients) [43]. Volumetric evaluation of residual tumor was not used in most of them, thus patients were stratified according to the subjective categories “total removal” and “subtotal removal”. Patients who had a GTR, based on the absence of contrast-enhancement on post-op MRIs, had a 61% chance of survival at 1 year after surgery, which was reduced to 19% at 2 years, and were 51% more likely to be free from disease progression at 1 year than those undergoing STR.

In a later retrospective study, Sanai et al. demonstrated a stepwise improvement in OS over 95% (*p* < 0.0001) [174].

The survival benefit resulting from resection of hyperintense FLAIR tumoral signal represent another relevant and debated issue. Recent studies have introduced the concept of “supramaximal resection”, which has been more frequently applied for low grade gliomas including resection of enhancing tumor together with non-enhancing GBM tissue, but the results remain controversial [160, 175]. Altieri et al. suggested that FLAIR-guided EOR does not correlate with patient survival reporting that an EOR > 96% was significantly associated with the prognosis despite a FLAIR-guided wide resection [176]. In line with this investigation, Mampre et al. [177] showed that postoperative residual FLAIR volume was not associated with recurrence and/or survival, neither in patients who underwent GRT of the CE portion of the tumor, nor in STR group. They also proved that CE residual tumor volume is more important than FLAIR volume in terms of recurrence and OS. Conversely, two other studies showed an additional survival benefit when at least part of the T2/FLAIR-hyperintense abnormality was resected [167, 170].

Although current literature strongly supports the role of EOR as independent predictor of OS, underling different survival benefit across the resective categories, recent studies evidenced that the absolute residual tumor volume might be prognostically more relevant than the proportion of removed tumor [37, 160, 169, 178].

That is, a high degree of resection in a large tumor could result in a greater residual tumor mass than a low degree of resection in a small tumor. In accordance to this consideration, the value of EOR achieved it does not express a direct measure of the residual disease burden, which in turn represents the postsurgical therapeutic target (i.e., radiation therapy and chemotherapy) [160, 169].

In 2022 the international RANO *resect* group published a new classification system for extent of resection in glioblastoma based upon both the relative reduction of tumor volume (in percentage) and the absolute residual tumor volume (in cm^3^) on postoperative MRI.

The author retrospectively analyzed the volumetric respective data in more than 1000 patients, founding that patients with “maximal CE resection” (class 2) had superior outcome compared to patients with “submaximal CE resection” (class 3) or “biopsy” (class 4). In addition, the authors demonstrated that a removal of non-CE tumor (≤ 5 cm^3^ residual non-CE tumor) beyond the CE tumor borders may translate into additional survival benefit [178].

Whether it is mainly the degree of EOR or glioma genetic signature to drive prognosis is yet to be defined. Interactions between molecular class and EOR subgroups has been an emerging topic of intense interest. Considering the importance of individual prognostic risk factors and genetic variability of gliomas, it is important to adopt analytic models to establish how these variable hierarchically interact with each other and how they impact survival in the complexity of clinical setting [8].

## Tools and strategies to maximize the resection

Over the past two decades, modern pre- and intraoperative imaging techniques, along with surgical tools and developments in monitoring techniques, have improved the potential to achieve a maximal safe resection of glioma [157, 175, 179]. Among neurosurgical centres, different imaging techniques and intraoperative tools are combined, resulting in a wide variety of surgical strategy protocols of proven value in maximizing the EOR [180]. Appropriate and wise integration of those techniques reduces intrinsic shortcomings combining specific strengths aimed at safe glioma resection.

### Surgical planning

Many different noninvasive methods may be used for preoperative planning to identify the relationship between glioma and eloquent areas, both at cortical and subcortical level [181].

Preoperative imaging techniques are useful in surgical planning and improve the preoperative communication of surgical risk to patients.

Functional Magnetic Resonance Imaging (fMRI) with Diffusion Tensor Imaging (DTI), fiber tractography and neuronavigated transcranial magnetic stimulation (nTMS) are widely employed for this purpose [182, 183]. In few centers, positron emission tomography (PET) and Magnetoencephalography (MEG) are also used, even though they are mostly applied in a research setting [184].

#### fMRI

Functional magnetic resonance techniques allow to establish a functional map of the eloquent regions involved in patients harboring brain tumors [185]. fMRI assesses brain activation by detecting modifications in blood oxygenation level by using the blood oxygen level-dependent (BOLD) contrast [186]. Task-based fMRI is used to localize non-invasively eloquent cortical areas [187]. Resting-state-fMRI is emerging as pre-surgical tool with automatic software for extraction of different networks at the whole brain level [188, 189]. In clinical setting, task-based fMRI compares BOLD signal changes while performing specific tasks to baseline conditions, based on the assumption that increased cerebral blood flow reflects neuronal and synaptic activity [190]. Its major limitation is that even minor alterations in neurovascular coupling, task execution, choice of coefficient correlations threshold (IV) or heterogeneity in data processing can degrade the quality and reliability of the fMRI results [190]. Basically, two groups of tasks and related cortical areas activations are commonly used: motor tasks and language-related tasks [186, 191], although paradigms for identification of visual [192] and sensory [193] areas have also been proposed.

Most studies suggested the feasibility and reliability of motor fMRI in presurgical planning [186], whereas the role of language fMRI is more debated [185, 194]. Indeed, the complexity of network connections related to language function, and the consequently heterogeneous tasks and post-processing techniques, may have a significant impact on the areas identified in language fMRI studies. This complexity and high variability is underlined in the literature reporting highly variable concordance rates, with sensitivity and specificity ranging from 59 to 100% and 0 to 97%, respectively [195, 196].

In patients affected by brain tumors, preoperative task-based fMRI has demonstrated to be a valid and highly sensitive tool for localizing eloquent cortical areas. Nevertheless, its prognostic role both in terms of reduced morbidity and improved oncologic outcome remains not definitively addressed and clarified.

#### nTMS

Navigated Transcranial magnetic stimulation is being increasingly used for presurgical planning of brain tumors located in eloquent areas [197, 198]. nTMS merges neurophysiological information with advanced imaging, thus providing a non-invasive preoperative mapping of functional cortical areas. nTMS overlaps the eloquent cortical sites to a 3D rendering of patient’s brain MRI, based on navigation, allowing for a customized planning, and anticipating the intraoperative responses of direct electrical stimulation [199]. In particular, nTMS consists in the application of a coil with an electrically induced magnetic field over the patient’s head. The magnetic field induces a modification of the neuronal excitability of the cortex, resulting in an excitatory or inhibitory effect that can be measured at a cortical level or recorded at the peripheral muscles [200]. Currently, nTMS excitatory parameters are used for mapping the primary motor cortex through its activation and the recording of motor evoked potentials at the peripheral muscles [201]. Conversely, specific inhibitory stimulation paradigms, based on repetitive nTMS, are used to map complex cognitive functions by evoking a “transient focal inhibition” to the underlying eloquent cortex during a specific task (e.g., object naming), hampering its correct execution. This result confirms the involvement of the inhibited area in the investigated cortical function [201], e.g., language [202]. Some evidences suggest its ability to map also other cognitive functions, including visuospatial skills and executive functions [203–206].

Several studies demonstrated that nTMS mapping of motor and language cortex correlates well with findings obtained by intraoperative direct cortical electrical stimulation [207–209], despite a slightly reduced sensitivity for language [210]. Moreover, several reports demonstrated that nTMS mapping is more reliable than fMRI in the identification of primary motor cortex [211–213], and that the use of nTMS improves motor outcome of patients operated for contrast-enhancing glioma in or close to motor cortex [214–216]. A recent meta-analysis concluded that nTMS motor mapping increases the EOR, improves neurological outcome, and enables a tailored surgical approach for motor-eloquent brain tumors [217].

Finally, nTMS motor mapping can be successfully combined with intraoperative sodium-fluoresceine-guided glioma resection, resulting in increased EOR compared to using fluoresceine alone [218, 219]. Evidences suggest that also nTMS-based language mapping may improve clinical outcome for those lesions located close to the language areas, especially in those not eligible to awake surgery [220–222].

#### Diffusion imaging with MR tractography

Currently, magnetic resonance tractography represents a unique tool to perform an *in-vivo* depiction of the anatomical course of main white matter fascicles. MR tractography is based on diffusion MR acquisitions that depict and quantify the anisotropic movement of water along white matter fibers; however, the accuracy of MR tractography is strictly correlated to the specific imaging acquisition protocol used [186]. In the clinical practice, the most used algorithms for image acquisition and reconstruction of white matter bundles are based on Diffusion Tensor Imaging (DTI), usually integrated in the most of the MR scanner and neuro-navigation stations. Therefore, tractography is commonly used for preoperative planning in order to identify the spatial relationship between lesions and surrounding white matter tracts [186, 223–225]. Nevertheless, tractography algorithms suffer from some bias, including the inter-operator variability in selecting anatomical landmarks for tract computation, and reduced accuracy in identification of crossing and ‘kissing’ WM fibers [226–228]. Therefore, new advanced dMRI models and processing algorithms have been developed to solve multiple fiber orientations and to capture complex fiber configurations, thus increasing the accuracy of tractography [229, 230]. In particular, new diffusion MR acquisition protocols such as high angular resolution diffusion-weighted imaging (HARDI) [231, 232] and new probabilistic algorithms such as Constrained Spherical Deconvolution (CSD) and q-ball imaging seem to be more accurate than standard DTI and DSI tractography for planning [233–236]. However, these approaches are less commonly available and require specific post-processing skills. Furthermore, nTMS-based seeding of the standard DTI reconstruction has been recently described. nTMS-based DTI fiber tracking, based on neurophysiological mapping of eloquent cortex, reduces inter-operator variability. Several studies documented a higher accuracy of nTMS-based DTI fiber compared to standard DTI tractography both for reconstruction of motor [183, 226, 237, 238] and language [239–241] tracts. Indeed, the implementation of preoperative nTMS-based tractography in brain tumor surgery resulted in the improvement of patients’ outcome [242–244].

More recently, fMRI-targeted tractography reconstructions of language tracts have been reported as an useful tool to depict the functional subcortical network underlying each fMRI task and the “high-risk subsets” of the subcortical bundles that should be spared during the surgical procedure [245].

Ultimately, by evaluating structural matter changes in combination with the preoperative functional and cognitive assessment, DTI could potentially represent a feasible predictive tool for patient counselling and risk assessment prior to surgery [246, 247].

### Intraoperative imaging

Different intraoperative technologies have emerged in recent years with undetermined comparative efficacy in optimizing EOR. An investigation review by Jenkinson et al. [248] provided low- to very low-evidence in single trial analyses and synthesis of results was not possible.

The effects of image-guided surgery on OS, PFS, and quality of life is demonstrated in large case series [60, 168, 248–254], but a functional comparison is poorly documented [251]. Network and traditional meta-analyses are generally not possible due to high risk of bias, heterogeneity of study population, and variable, not standardized outcome evaluation.

An increasing number of new technologies have been routinely used intraoperatively to enhance tumor visualization and guide the resection. Thus, several biomedical engineering devices aimed at optimizing performance during oncological neurosurgery interventions, such as neuro-navigation systems, intraoperative CT (iCT) and MRI (iMRI), and iUS are available nowadays [175, 180]. Furthermore, gliomas surgery is implemented thanks to the use of fluorophores that allow a better distinction of tumoral tissue from healthy brain tissue compared to microscopic view under white light. The purpose of these methods is to obtain maximal EOR, while preserving neurological functions, especially in cases of neoplasms located in eloquent areas.

#### Neuronavigation

Among modern tools for resections, neuronavigation systems represent the most used intraoperative tool used by neurosurgeons. Based on preoperative imaging, generally MRI or CT scans, with functional sequences if available, neuro-navigation allows preoperative depiction of the lesion and surrounding anatomical and eloquent structures and guides intraoperative mapping and tumor resection.

Neuronavigation provides intraoperative orientation to the surgeon, helps in planning a precise surgical approach to the targeted lesion, and defines the surrounding neurovascular structures. It has become a mainstream component of the neurosurgical armamentarium and its use leads to improved surgical confidence, accurate bone flap placement, reduced craniotomy size, and anatomical orientation [51, 52]. Incorporation of the functional data provided by fMRI, MR tractography, magnetoencephalography (MEG), or DES atlases with neuronavigation helps to avoid eloquent areas of the brain during surgery [21, 255]. Main limitations of this device are errors during registration, brain shift and local tissue deformation, which reduce the accuracy of real-time neuronavigation [256]. Moreover, integration of tractography into neuronavigation has still a limited value to identify subcortical tracts [159].

#### Intraoperative MRI

The advantages of using intraoperative MRI (iMRI), compared to the guidance provided by conventional neuronavigation systems, were highlighted in a recent meta-analysis [62]. iMRI was found to be associated with higher GTR rates compared to neuro-navigated procedures, whereas a substantial difference between the two techniques was not found regarding quality of resection and surgical time. With regards to impact on outcome, use of iMRI has been associated with increased PFS compared to neuronavigation, although OS was similar in the two groups [62].

Moreover, a recent study verified the implementation of the novel Black Blood (BB) imaging technique for intraoperative identification of lack/presence of contrast-enhancing tumor residuals and better delineation of the boundaries of contrast-enhancing malignant tissue. BB imaging is not inferior to conventional turbo field-echo (TFE) imaging for EOR assessment, nonetheless it may significantly reduce iMRI scanning time, whilst increasing diagnostic confidence. Furthermore, given the better depiction of contrast-enhancing tumor residual spread and borders, BB imaging may help improving the degree og glioma resection [61]. Unfortunately, high costs related to structural changes in the operating room to bear the burden of machinery, capital equipment expenses, suite constructions and renovations, and the associated personnel and maintenance costs have limited, to date, the spread of intraoperative MRI.

#### Intraoperative CT scan

Shalit et al. first described the use of intraoperative CT (iCT) scan for brain tumors in 1979 [257], which represents a further technological improvement introduced in neurosurgery. Several studies have reported its use, documenting its effectiveness and applicability to neurosurgical interventions for different types of lesions [258, 259].

The iCT role in maximizing the EOR has been recently investigated [57]. iCT helped to verify EOR, identify and resect tumor residue also in multifocal tumors. Compared to iMRI, iCT represents a feasible and effective alternative for intraoperative update of neuro-navigation system, providing real-time images, based on its faster execution times. The possibility of contrast administration increases the accuracy of definition of pathological tissue.

#### Intraoperative ultrasounds (iUS)

iUS is a real-time, accurate and inexpensive imaging method for optimizing EOR in neurosurgical interventions. The main issues of intraoperative iUS use are the choice of the appropriate probe and the interpretation of US images in the three orthogonal planes (axial, sagittal and coronal plane). The difficulties of recognizing regional anatomy can be overcome by practice: training on large number of cases is important to obtain valuable real-time information [249]. Moreover, the possibility of MR and CT-US imaging fusion for real-time neuronavigation improves the learning curve by help interpreting iUS imaging with the guide of more familiar CT and MR images [260].

A meta-analysis conducted on 790 articles published from 2005 to 2016 documented how use of iUS with contrast administration in glial tumor resection, despite being an operator-dependent method, allowed GTR in 77% of cases and was associated with increased PFS and OS [253].

There are currently no standardized protocols or validated quantitative data guiding the use of iUS. A retrospective study based on quality of resection of intracranial tumors of different types showed that iUS tends to offer dynamic imaging able to correct errors due to anatomical distortion (brain shift and local tissue deformation) in real-time, which conversely limits conventional neuro-navigation systems based on imaging studies performed in the pre-operative phase. Contrast administration provides useful data for intra-operative diagnosis, tissue differentiation and a real-time evaluation of EOR [58]. Further prospective studies are needed to standardize the role of iUS in a neurosurgical setting.

#### Fluorescence: 5-aminolevulinic acid (5-ALA)

Fluorescence induced by 5-ALA allows, by using a metabolic tracer, intraoperative distinction between healthy tissue and tumor at infiltration margins, thus guiding glioma resection [65, 158, 261, 262].

A multicenter, randomized phase III study on fluorescence guided surgery (FGS) using 5-ALA showed a more complete resections of tumors in enhancing-glioma patients and better patient outcomes than with conventional microsurgery. Complete resection of the enhancing portion of newly diagnosed GG4 occurred in 65% of patients using 5-ALA versus 36% in those assigned to conventional surgery white light group (difference between groups 29% [95% CI 17–40], *p* < 0·0001). In addition to higher rate of complete resections, overall progression-free survival at 6 months (PFS-6) was also significantly greater with 5-ALA FGS (41.0% [32·8–49·2] vs. 21.1% [14·0–28·2]; *p* = 0.0003) [168].

In several studies, it has been shown that tumor resection carried out with the aid of 5-ALA fluorescence is associated with a greater rate of GTR and an increase in PFS [251, 254].

Glioma surgery, especially for lesions harboring in or close to eloquent areas, should respond to two different needs: efficacy in terms of surgical radicality and preservation of neurological function. In a single-center retrospective study [263], the influence on survival with 5-ALA fluorescence for supratentorial gliomas in eloquent areas was evaluated. With the same EOR and OS rates, PFS was significantly longer in 5-ALA group than in the control group of patients undergoing the white light procedure.

A multimodal approach conjugating 5-ALA for intraoperative visualization of tumor tissue with neurophysiologic cortico-subcortical mapping and monitoring of eloquent areas with fMRI and fiber tracking integrated in neuronavigation system has proven to be useful in improving safety and preserving neurological functions during glioma surgery in eloquent areas, overcoming the advantages obtained with single methods [53].

In the research setting, intraoperative tissue sampling 5ALA guided has been recently proven to be useful in providing insights into the heterogeneity of the spatial glioma microenvironment, underlying the importance to extend the EOR beyond the fluorescence tumor borders when functionally possible [264].

#### Fluorescence: sodium fluorescein (FLCN)

Recent evidence suggests that intravenous administration of FLCN at induction of anesthesia at a dose of 5 mg/kg represents an important contribution toward maximal resection of GBMs [74, 265, 266].

Intraoperative guidance by FLCN allows to predict histopathological alterations both in areas with contrast enhancement and, with a positive predictive greater than 96%, also at the infiltration non-enhancing margins [73].

FLCN is an easily, available, bio-safe and cheap fluorescein dye FDA approved [65, 267].

The presumed mechanism of action is a passive staining of the extracellular space in areas with disrupted BBB, then it corresponds to gadolinium uptake on magnetic resonance imaging. It implies that the fluorescent area corresponds manly to enhancing nodule seen at pre-operative MRI T1 after gadolinium administration. Some investigations showed that a SF enhancement could extend beyond gadolinium contrast-enhancing regions, probably because of the smaller molecular weight of SF that allows its diffusion trough the damaged BBB.

SF has, however, no specificity for tumor cells in comparison with 5-ALA. Different fluorescence patterns within the tumor are not detectable. The identification of tumor border is thus more difficult and less precise.

Despite these limitations, evident especially in the early use of fluorescein in the FGS, the progressive and extensive use of SF in has led to better exploit all its potentials. Neira et al. demonstrated indeed that intraoperative SF staining correlated with histopathological alteration in both contrast enhancing and non-contrast enhancing regions, with a PPV greater than 96% in non-contrast-enhancing regions, suggesting that SF can be used as a visual marker for glioma resection in both regions of GBM [73].

### Brain mapping and neurophysiological monitoring

Although preoperative imaging modalities can facilitate surgical planning, direct cortical and subcortical electrical stimulation remains the gold standard for localizing brain function [268].

More than 50% of GG4 developed near or in eloquent areas. Therefore, when resecting GGS the onco-functional principle of maximal safe resection has to be pursued. In this clinical setting, DES allows the surgeon to prevent damage to eloquent cortical and subcortical areas during re-section, maximizing the EOR in compliance with quality of life preservation [47].

There is compelling evidence that glioma resections using DES are associated with fewer late severe neurologic deficits and more extensive resection [44, 45, 269–271].

Intraoperative neurophysiology offers various stimulation modalities, which efficiency is based on the ability to recognize essential sites with the highest possible resolution.

The bipolar stimulation is the most used technique for cortical and subcortical brain mapping [44, 46, 269, 272, 273]. A growing body of evidence are currently supporting the use of high-frequency monopolar stimulation in glioma surgery [274–276]. The integration of two stimulation paradigms have been recently proposed for intraoperative guidance of motor tumors removal: the 60 Hz-technique [low frequency (LF)] and the pulse-technique [high frequency-(HF)], delivered by bipolar or monopolar probe respectively. The integration of stimulation modalities with clinical context enhances the extent and safety of resection [272].

In addition, motor evoked potentials (MEPs) can be applied to monitor motor function during resection. This may be performed transcranially (tcMEP) or by direct cortical stimulation via a grid or strip electrode (dcMEP) [268, 277].

The above techniques can be used independently or in various combinations. Gogos et al. demonstrated that transcranial and direct cortical MEP monitoring combined with bipolar and monopolar stimulation resulted in improved localization of functional tissue and low rates of transient and permanent deficits [277].

### Awake surgery

Brain mapping techniques and awake surgery (AS) represent the gold standard in low grade glioma (LGG) resection [278, 279], whereas the value of AS for GG4 is poorly investigated [20, 48, 49]. A recent meta-analysis based on 53 studies, including 9102 patients, demonstrated that AS and direct electrical stimulation (DES) resulted in an effective surgical strategy even for GG4 in eloquent areas. This intraoperative technical combination provides OS a lower rate of postoperative complications and a higher percentage of cases with gross total resection, which implies a consequent survival benefit [47]. This technique is usually adopted in younger patients, with circumscribed lesions. Recent volumetric studies demonstrated a negative impact of postoperative T2/FLAIR residual tumor on GG4 OS [160, 280, 281]. Based on these findings, AS may represent an effective surgical approach to safely maximize resection beyond contrast-enhanced tumor area in highly selected cases (collaborative patients without preoperative language deficits harboring lesions in or close to language areas amenable of gross total resection, in the absence of intracranial hypertension). In addition, AS could be useful in association with 5-ALA to detect functions in fluorescent tissue guiding a safe resection [48]. Future perspective investigations are needed to determine efficacy and outcomes of AS in GG4 patients.

## Intraoperative treatment options

### Carmustine wafers (CWs)

In 2003, the intraoperative treatment with Carmustine Wafers (CWs) implantation [marketed as Gliadel, biodegradable copolymers discs impregnated with the alkylating agent (Bis-ChloroethylNitrosoUrea: BCNU)], for newly high grade glioma was introduce as a therapeutic bridge between the surgical resection and Stupp Protocol onset. The clinical rational of its development was based on the possibility to locally interfere with the potential tumor re-growth in the proximity of the original tumor site [77, 80, 81].

Different studies demonstrated a promising results in terms of PFS, without detecting a significant survival advantage [79, 282, 283].

A phase 3 study conducted in 14 countries, including the United States, Germany, France, the United Kingdom, Scotland, Finland, and Israel, suggested a prolongation OS in newly diagnosed patients with malignant glioma who received CWs implants. The median OS was 13.9 months for the CWs group and 11.6 months for the placebo-control group (logrank *P* = 0.03 stratified by country) [283].

In a later investigation, Pallud et al. designed a largest case-matched analyses on CW implantation efficacy, founding a survival advantage of only 2 months in the implantation group [79].

The elevated costs in addition to the precluded enrolment of patients in subsequent clinical trials, because the use of CW could give rise to confounding results, have led to a gradual abandon of it use after an initial enthusiasm. In addition, in several retrospectives studies reported a serious CWs related toxicity, resulting in a delay or precluded Stupp protocol [77, 80].

For all the reasons mentioned above, in newly diagnosed high grade glioma, CWs implantations should not be considered as first-line therapeutic option.

### Intraoperative radiotherapy

Adjuvant radiotherapy is considered standard of care in brain malignant gliomas treatment. Recently, in analogy with other cancers, intraoperative radiotherapy (IORT), has been proposed with the aim to provide a boost to standard-of-care external beam radiotherapy (EBRT), in both recurrent and newly diagnosed brain gliomas. Some experiences with IORT have been published lacking in suggesting significative improvement in OS and PFS in both newly diagnosed and recurrent gliomas [284, 285]. A Multicenter Randomized Phase III Trial on INTraoperative RAdiotherapy in Newly Diagnosed GliOblastoma Multiforme (INTRAGO II) is ongoing and will stop recruitment on December 2023. Recently, a pooled analysis has been published suggesting improved efficacy and safety compared to historical control of low energy intraoperative X-ray for newly diagnosed glioblastoma [286].

## The role of biopsy

Stereotactic image-guided brain biopsy (SB) is the procedure of choice when an oncologically meaningful surgical resection is not feasible, as in case of deep-seated or multifocal tumors, or if the patient has considerable comorbidities increasing the risk of perioperative morbi-mortality [287, 288]. In such cases, SB is a safe and effective diagnostic technique, with a diagnostic yield of approximately 90% [287, 289, 290].

SB could be performed through frame-based or frameless techniques. Even though frame-based techniques have been considered the “gold standard” for SB as the rigid frame provides excellent targeting precision SB by frameless techniques, also conducted with the assistance of robotic devices, mainly for brainstem or small deep lesions, has gradually replaced the previous one [289, 291]. They showed equal diagnostic yield, in addition to significantly shorter surgical time and less discomfort reported by patients [292].

It is important to note that the tissue size must be adequate to provide MGMT analysis, which could be useful in determining the potential response of chemotherapy treatment, especially in the elderly [293].

Recent coupling with intraoperative MRI systems provides a real-time feedback on targeting [294]. However, no significant differences in diagnostic yield were found compared to neuronavigation on preoperative imaging [290].

Lately, various strategies have been devised to best target the significant portions of the lesions, to enhance the chances of obtaining a clear diagnosis, merging anatomic MRI with multimodal imaging including MRI perfusion and spectroscopy [295, 296] and PET-CT [297, 298].

Perfusion-weighted imaging such as dynamic susceptibility contrast (DSC) MRI measures cerebral blood volume (CBV), which correlates with microvessel density and area. Magnetic resonance spectroscopy (MRS) can detect alterations of metabolite concentrations within the tumor. PET-CT can provide information about biology, differential diagnosis, delineation of tumor extent for surgical and RT planning, which can also be useful in post-treatment surveillance (progression vs pseudoprogression) [237].

Despite being rapid and minimally invasive, SB still poses some risks. Mortality rates reported from large populations studies range from 0.6 to 3.8% [288, 289, 299–302], generally consequent to brain edema or haemorrhage. Deep-seated lesions were found to be associated with higher risk of overall post-operative death, whereas frontotemporal lesions and lymphomas were associated with an increased probability of haemorrhage leading to death [289].

Complications after SB range from 7.4% to 13% and include symptomatic haemorrhage, seizures, infections, change of mental status, and new neurological deficits [289, 299, 302]. Diabetes mellitus and deep-seated lesions appear to increase the risk [302]. Post-operative haemorrhages, in particular, may occur in 7–59.8% of patients; however, only 3.4–5.9% are symptomatic [303, 304]. Biopsy-related haemorrhage risk is higher with hydrocephalus, brain edema, and advanced age [305]. Tumor seeding along the course of the biopsy needle is a rare complication that has been described in the literature [306, 307].

Navigate guided stereotactic systems are recently introduced in routinely neurosurgical practice. Different neuronavigated systems, such as intraoperative CT scan, MRI or US tools, are progressively applied to a comprehensive range of neurosurgical procedures, including brain tumors surgery. These systems allow a minimally invasive surgical exposure and provides instant and continual navigational information during surgery.

Different studies have shown that these navigate guided systems can reduce operative time, increasing the neurosurgeon’s confidence with the anatomic structures of the brain and improving the surgical safety of the biopsy [308].

Finally, deep brain biopsies with accurate tumor localization are made possible by combined use of computerized imaging and stereotactic devices [309].

## Second surgery at tumor recurrence

When a signal alteration is documented in follow-up MRI images post-adjuvant therapy, a differential diagnosis should be considered within progression and pseudoprogression. Multi-disciplinary discussion and advanced MRI images could define the real glioma recurrence [310, 311]. It remains unavoidable even if improvements in oncological and surgical treatments may delay this event. The indication to a second surgical operation for HGGs is controversial, in particular regarding selection of patients. In the literature, several variables have been considered to support surgical decision. Reoperation, especially when associated with a favourable preoperative Karnofsky Performance Status (KPS) at recurrence, was regarded as statistically significant variable for improved survival [90, 92, 312–315]. A greater EOR at 1st and 2nd surgery correlate with longer OS 88–92. Nevertheless, regarding surgical variables, conflicting results have been reported in the literature. Some authors found no significant effect of surgery on survival, or no difference between gross-total resection (GTR) and partial resection [316, 317]. However, there are growing evidences that EOR > 98% at second surgery greatly improve the OS [318, 319]. GTR at second surgery seem to correlate with better OS and post-operative KPS [88–92]. Finally, a benefit is reported in patients with higher KPS score at diagnosis, a greater EOR and initial diagnosis of WHO grade III. About one-third of patients with HGG may be eligible for salvage surgery at the time of progression [320].

Role of systemic therapies at time of recurrence is still debated and standardized protocols are missing, even though recently regorafenib has been included in the NCCN (National Comprehensive Cancer Network) 2021 guidelines as a preferred regimen for recurrent glioblastoma (GB) [321]. Despite randomized trials showing that salvage TMZ before radiation therapy or anti-PD1 immunotherapy before surgery prolong disease-free survival in patients with recurrent GG4 [322–324], combination of surgery and adjuvant therapies with TMZ, fotemustine, carmustine, irinotecan or low-dose fractionated RT has been reported as the best treatment strategy in terms of survival [317]. Administering neoadjuvant therapy before reoperation seems to correlate with poorer KPS, while adjuvant therapy after reoperation is associated with a better OS.

Surgery for recurrent HGG should be carefully evaluated in case of treatment with anti-angiogenic agents (bevacizumab). As a matter of fact, patients receiving bevacizumab are more likely to develop wound complications, CSF leak, infection and osteomyelitis. In case of second surgery for progression during bevacizumab treatment patients exhibit poor prognosis for the in-creased risk of perioperative complications [325]. If surgery is considered mandatory, re-operation should be delayed for at least 4 weeks after discontinuing bevacizumab [326].

Regarding patient factors, age < 70 at recurrence confirms as crucial favourable factor [327–329], correlating with OS > 6 months following recurrence. However, KPS at time of recurrence appears to be a stronger factor than age [330]. Indeed, especially if ≥ 90, it correlates positively with both OS and post-op KPS.

Regarding histological factors, IDH1 mutation is associated with longer survival and improved clinical outcome [331–333]. Nevertheless, despite a globally better clinical course, it is not clear if a correlation with second surgery exists. On the contrary ATRX and PTEN inexpression at first surgery correlates with better KPS and PO.

Nevertheless, these evidences require randomized controlled trials to be confirmed and to support the development of guidelines on management of GG4 recurrence. Surgical indications are still debated, although most studies report improved survival. An adequate patient selection is crucial to achieve the most satisfactory clinical outcome. Given the results of our multicentre retrospective study, a high KPS at recurrence and a GTR following second surgery are pivotal to survival gain and preservation of a high-performance status. Another issue to be explored is the role of early second-look surgery for patients with unintentional incomplete glioblastoma resection detected by early postoperative MRI. Scattered evidence exists of an increased EOR without additional neurological deficit, portending Re-do surgery as a feasible strategy to increase the rate of complete resections in glioblastoma patients [334].

## Surgery in elderly glioma patients

GG4 is the most frequent brain tumor in elderly patients (over 70 years), with an incidence rate of 17.5 per 100,000 [335] a poorer prognosis is associated with older age [336], comorbidities, and an intrinsic most aggressive behaviour due to clinical and genetic features [337]. The median survival of elderly patients is approximately six months [338–341], as a result of a debated strategy regarding the optimum management.

Although elderly population is constantly increasing, because of the frailty of elderly and the well-known unfavourable behaviour of GG4 lesions, many neurosurgeons tend to avoid aggressive surgical interventions in this population because of an increased risk of perioperative complications [337, 342].

In recent years, however, there is increasing evidence suggesting that advanced age alone should not necessarily preclude optimal resection followed by adjuvant RT and CT in these patients 337, 342–346.

Standard of care for newly-diagnosed GG4 in elderly patients consists, if feasible, in surgical resection followed by a short course of RT with concomitant and adjuvant TMZ [345].

A latter multicentre investigation demonstrated that surgery can be considered as a first therapeutic option in the workflow of elderly patients, especially when the preoperative estimated EOR is greater than 80% [342].

The OS in elderly patients affected by GG4 is similar to that of younger adults, if factors such as medical comorbidities, effects of general anaesthesia, and vulnerability to postoperative complications, such as delirium, do not overweight the expected clinical benefit. Therefore, a tailored surgical treatment should be carefully planned, according to tumor size and location, patient comorbidities, and preoperative estimation of achievable EOR [347–352]. A thorough evaluation and patients selection are essential to obtain both a favourable survival and functional benefit [337, 342].

## Conclusions and future directions

A growing number of evidences support the role of EOR as independet predictor o OS in GG4 patients. The ongoing development of novel  intraoperative techniques and strategies for a precise real-time identification of peritumoral functional pathways enables surgeons to maximize EOR minimizing the post-operative morbidity.

Extending the resection according to T2 or FLAIR tumor boundaries implies that functional areas will be encountered. In this clinical setting, DES and brain mapping remain the gold standard technique to detect and monitor the functional networks both at cortical and subcortical level.

Functional MRI and tractography may support the preoperative planning and assist surgeons in selecting the safest surgical approach.

Future prospective randomized clinical trials are needed to compare the influence of the different intraoperative image-guided glioma resection techniques (i.e., NN versus iUS, versus iUS combined with 5-ALA and ore intraoperative neurophysiological monitoring).

The 2021 WHO classification has introduced important changes in each taxonomic category poorly investigated in clinical trials. Future integrative analyses, combining the molecular class according to the 2021 WHO classification and the degree of resection achieved in different MRI sequences, may thus allow a thorough detection of patients with different prognosis, implying a redrawing of the current investigations.

## Data Availability

Data regarding screeing process are available upon request.
